# Chronic Opioid Use and Endocrine Disruption in Women: Mechanisms, Life-Course Vulnerabilities, and Reproductive Health Implications

**DOI:** 10.3390/jox16030106

**Published:** 2026-06-07

**Authors:** Doonyah Alucozai, Elizabeth Kwo

**Affiliations:** 1 Department of Anatomy, Cell Biology, and Physiology, Indiana University School of Medicine, Indianapolis, IN 46202, USA; 2 Department of Medicine, Harvard Medical School, Boston, MA 02115, USA; lizkwo@mail.harvard.edu

**Keywords:** opioids, endocrine disruption, women’s health, reproductive toxicology, xenobiotic exposure

## Abstract

Chronic opioid use disrupts the female endocrine system, affecting the hypothalamic–pituitary–gonadal (HPG), hypothalamic–pituitary–adrenal (HPA), and hypothalamic–pituitary–thyroid (HPT) axes. In women, these disruptions manifest as menstrual irregularity, infertility, early menopause, reduced bone mineral density, adrenal insufficiency, and altered mood and sexual function. Despite the magnitude of the opioid epidemic and its impact on women of reproductive age, the existing evidence base is overwhelmingly male: across the major studies of opioid-induced endocrinopathy reviewed here, 99.5 percent of participants were male. Sex-specific mechanisms, prevalence estimates, and clinical thresholds therefore remain poorly defined, and current guidelines do not adequately address the reproductive, skeletal, and adrenal consequences of chronic opioid exposure in women. This review synthesizes available human and preclinical evidence on opioid-induced endocrine dysfunction in women across the lifespan, distinguishing established findings from hypotheses extrapolated from male or animal data. We propose a practical framework for routine endocrine screening of HPG, HPA, and HPT axis function, bone health, and fertility, and outline the roles of relevant specialties in multidisciplinary care. Available evidence suggests a substantial risk of endocrine dysfunction in women on chronic opioid therapy, but the precise prevalence remains unknown. Sex-sensitive research, guidelines, and routine screening are urgently needed to close this gap.

## 1. Introduction

### 1.1. The Opioid Crisis and Sex-Specific Vulnerabilities 

The opioid epidemic is one of the defining public health crises of the 21st century, and women have borne a disproportionate share of its recent escalation. Between 1999 and 2021, drug overdose deaths among all women in the United States increased by approximately 480%, with overdose rates rising more steeply in women than in men over recent decades [1]. This escalation reflects both the rise in pharmaceutical opioid prescribing and the growing contamination of illicit drug supplies with manufactured fentanyl and its analogs [2]. Mortality, however, is only part of the problem. Chronic opioid use carries a burden that reaches far beyond pain perception and addiction pathways. The endocrine consequences remain particularly understudied. Opioids act as xenobiotic agents that disrupt neuroendocrine homeostasis at multiple levels, yet this toxicity is largely absent from clinical discourse and medical education [2,3].

Women face particular vulnerability for three reasons, each of which compounds the others. The first is anatomical. Opioid receptors are densely expressed throughout the hypothalamus, pituitary gland, and ovaries, and female reproductive endocrinology depends on precise hormonal regulation that chronic opioid exposure readily disrupts [4]. The second is developmental. Adolescence, reproductive maturity, pregnancy, and perimenopause are distinct windows of endocrine plasticity in which xenobiotic insults may produce lasting effects [5,6]. The third is pharmacological. Chronic opioid use overlaps with fertility, pregnancy, and hormonal contraception, generating pharmacokinetic and pharmacodynamic interactions that remain poorly characterized [7].

### 1.2. Opioid-Induced Endocrine Dysfunction

The endocrine effects of acute opioid exposure, particularly opioid-induced hypogonadism and secondary adrenal insufficiency, have been recognized in clinical practice for decades [8,9]. However, the mechanisms, prevalence, and long-term consequences of chronic opioid-induced endocrine dysfunction in women remain poorly understood compared to men. In the most comprehensive meta-analysis to date, de Vries et al. [10] reported that the vast majority of pooled participants were male. Women comprised less than 0.5% of the total sample. The reported 63% hypogonadism prevalence is therefore a number drawn almost entirely from men and cannot be reliably generalized to women [10]. Clinical recognition of opioid-induced endocrine dysfunction in women has also been limited by diagnostic challenges. Symptoms of opioid-induced hypogonadism, including fatigue, mood disturbance, sexual dysfunction, and decreased bone density, overlap extensively with depression, anxiety, and other comorbidities. This overlap creates opportunities for symptom misattribution and delayed recognition [11].

### 1.3. Opioids as Xenobiotic Endocrine Disrupters

**Figure 1:**
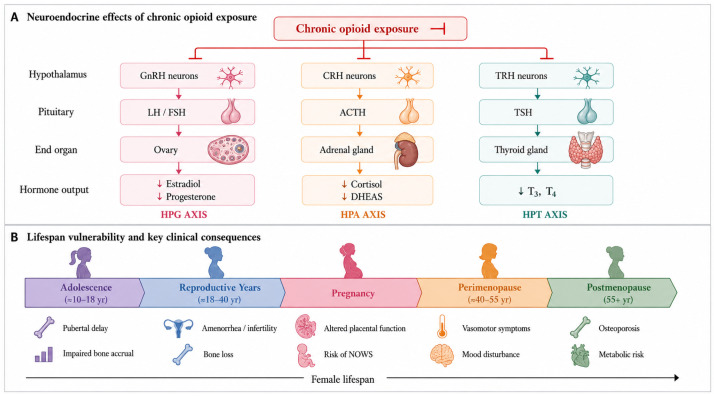
Overview of opioid-induced endocrine disruption in women. (**A**) Chronic opioid exposure suppresses hypothalamic GnRH, CRH, and TRH neurons, with downstream inhibition of pituitary and end-organ hormone output across the hypothalamic-pituitary-gonadal (HPG), hypothalamic-pituitary-adrenal (HPA), and hypothalamic-pituitary-thyroid (HPT) axes. Blunt-ended arrows (⊣) denote inhibition and downward arrows (↓) indicate decreased hormone levels. (**B**) Endocrine vulnerability and representative clinical consequences across the female lifespan, from adolescence through postmenopause, illustrating that the clinical expression of opioid-induced endocrinopathy differs by reproductive stage. Together, the panels convey that chronic opioid exposure produces a coordinated, multi-axis suppression of female endocrine function whose manifestations vary across the lifespan. DHEAS, dehydroepiandrosterone sulfate; NOWS, neonatal opioid withdrawal syndrome; $T_{3}$, triiodothyronine; $T_{4}$, thyroxine.

The xenobiotic framework, the study of foreign chemical substances found in an organism that is not normally produced or expected to be present, provides a useful lens through which to understand opioid effects on endocrine homeostasis [3]. Both exogenous opioids (pharmaceutical and illicit) and endogenous opioids (endorphins, enkephalins, dynorphins) act as signaling molecules. They produce a wide range of effects on the neuroendocrine system [3,4]. When the exposure becomes chronic, exogenous opioids lead to sustained activation of opioid receptors in areas like the pituitary and hypothalamus. This can disrupt the feedback mechanisms that regulate hormone secretion [9,10]. Beyond that, some opioids and their metabolites can bind to other targets or accumulate in endocrine tissues, for example, producing effects beyond typical opioid receptor signaling [3]. Figure 1 summarizes opioid action across the hypothalamic–pituitary axes, the resulting biochemical changes, and the characteristic clinical consequences observed across the female lifespan.

## 2. Mechanisms of Opioid-Induced Endocrine Disruption

### 2.1. Opioid Receptor Distribution and the Hypothalamic–Pituitary Axis

Opioid receptors exist in four main classes: mu (μ), delta (δ), kappa (κ), and nociceptin receptors [12]. Among these subtypes, mu receptors are most abundant in neuroendocrine tissues and are the primary targets of clinically used opioid analgesics [3]. High densities of mu opioid receptors are expressed in the hypothalamus, specifically in the medial preoptic area, ventromedial hypothalamus, and arcuate nucleus. These distributions are best characterized in rodent and non-human primate models. Human evidence derives primarily from post-mortem immunohistochemistry and PET imaging studies, which are consistent with the animal literature but less spatially resolved [3,4]. These regions play a central role in regulating gonadotropin-releasing hormone (GnRH) secretion, which acts as the master regulator of the HPG axis [4,9]. Delta and kappa receptors are also found in hypothalamic tissues, though they are generally less abundant than mu receptors. That said, kappa receptor signaling may still play an important role in stress-responsive neuroendocrine pathways [3]. The anterior pituitary gland contains opioid receptors on both gonadotroph and corticotroph cells. These provide sites for direct opioid modulation of luteinizing hormone (LH), follicle-stimulating hormone (FSH), and adrenocorticotropic hormone (ACTH) secretion [10]. Additionally, opioid receptors are expressed in the ovary, where they may influence local steroid production and ovulation [4].

### 2.2. Opioid-Induced Suppresion of GnRH and the HPG Axis

Chronic opioid exposure is thought to suppress GnRH secretion through several proposed, partially overlapping mechanisms. The majority of these mechanisms have been characterized in rodent and non-human primate models, with corroborating but less mechanistically detailed human evidence [3,9]. First, mu opioid receptor activation in the hypothalamus directly suppresses GnRH neuronal activity [3]. This can happen through GABA-mediated hyperpolarization of GnRH neurons, along with a reduction in excitatory glutamatergic input [9]. Second, opioids can boost GABA-driven inhibition and dampen glutamatergic excitation, resulting in an overall suppression of the GnRH pulse generator. Lastly, chronic opioid exposure may disrupt how GnRH neurons respond to upstream regulators like kisspeptin and the broader kisspeptin/neurokinin B/dynorphin (KNDy) network, which together drives pulsatile GnRH release [13,14].

Once GnRH secretion is suppressed, the effects spread throughout the HPG axis. Lower GnRH pulse frequency and amplitude reduce pituitary secretion of LH and FSH, which results in hypogonadism characterized by low circulating estradiol in women [8,15]. The timing and severity of this suppression are dose-dependent, meaning higher opioid doses produce stronger and potentially long-lasting endocrine effects [10,11].

More importantly, women may be especially vulnerable to opioid-induced HPG suppression. For one, the female HPG axis runs on episodic GnRH pulses at a lower frequency than in males, which may leave it more sensitive to opioid-induced disruptions [4]. Estrogen also boosts opioid receptor expression in the hypothalamus, creating a feedback loop in which estrogen withdrawal from opioid-induced hypogonadism can actually perpetuate the dysfunction [3]. Finally, the cyclical nature of the menstrual cycle adds another layer of complexity. Opioids can disrupt the mid-cycle LH surge, ovulation, and the hormonal transitions that define different cycle phases [16].

### 2.3. Opioid Effects on the Hypothalamic Pituitary Adrenal Axis 

The HPA axis responds to opioids quite differently than the HPG axis does. While acute exposure produces variable effects [3], chronic opioid use reliably dysregulates this system, manifesting as blunted HPA responsiveness to stressors [11]. This effect manifests clinically as hypocortisolism affecting 15% to 24% of chronically exposed patients [10,17], with higher prevalence in studies employing more rigorous assessment methods.

The mechanisms underlying opioid effects on HPA function appear to be multifactorial, though most mechanistic insights derive from rodent models, whereas human data remain limited [3,18]. Opioid receptors in the paraventricular nucleus (PVN) of the hypothalamus modulate HPA axis tone. The PVN is the principal site of corticotropin-releasing hormone (CRH) production. Mu opioid receptor activation suppresses CRH neuronal activity, potentially through enhanced GABAergic inhibition [3,9]. Additionally, opioids may enhance the activity of endogenous opioid peptides (β-endorphin) produced in the anterior pituitary, which can feed back to suppress CRH secretion. Over time, chronic opioid exposure can lead to downregulation of CRH receptors and blunting of the ACTH response to CRH, contributing to reduced cortisol production [11].

For women, it seems that HPA axis dysregulation carries particular clinical weight. The menstrual cycle modulates HPA axis reactivity, with cortisol stress responses varying across cycle phases. Opioid-induced disruption of menstrual cyclicity may therefore compound and obscure HPA dysfunction in ways that standard morning cortisol measurements fail to capture [16]. Moreover, adrenal androgens, particularly dehydroepiandrosterone sulfate (DHEAS), contribute proportionally more to total circulating androgen stores in women than in men, given the absence of testicular testosterone production. Opioid-induced adrenal suppression may therefore produce a dual burden in women: glucocorticoid insufficiency and androgen depletion. This combination is not adequately captured by HPG-axis screening alone. It is thought to contribute independently to diminished libido, fatigue, and mood disturbance [15,19].

### 2.4. Opioid Effects on the Hypothalamic Pituitary Thyroid Axis

The HPT axis represents another important neuroendocrine system affected by chronic opioid exposure, though the evidence base is less robust than for HPG and HPA effects. Evidence for opioid effects on the HPT axis seems to be mixed. While some studies document lower free thyroxine (T4) and higher TSH in chronically exposed individuals, these findings have not been consistent across pooled analyses [10]. The mechanism is thought to involve opioid suppression of TRH in the hypothalamus, however this remains incompletely characterized [3]. Despite inconsistent population level findings, HPT effects remain clinically relevant for women specifically. Women are 5 to 10 times more likely than men to have autoimmune thyroid disease. Even mild opioid-induced thyroid disruption can therefore carry meaningful consequences in an already vulnerable population. Thyroid hormones also play essential roles in menstrual regularity, ovulation, and pregnancy, further underscoring the reproductive relevance of HPT axis changes in this group [20].

### 2.5. Metabolic Consequences of Opioid-Induced Endocrine Disruption

Beyond direct effects on hormone-producing tissues, opioid-induced endocrine disruption produces widespread metabolic consequences. Estrogen deficiency contributes to insulin resistance, visceral adiposity, and disordered glucose metabolism, collectively raising the risk of metabolic syndrome [2]. At the same time, opioid-related HPA suppression reduces cortisol availability, which is itself necessary for normal glucose and lipid regulation, compounding the metabolic burden already created by hypogonadism [11]. Chronic opioid use may also directly affect appetite-regulating hormones such as ghrelin and leptin, though the evidence for this remains inconsistent and requires further investigation [2]. Together, the convergence of HPG suppression, HPA dysregulation, and potentially altered appetite signaling places women with chronic opioid exposure at considerable and compounding metabolic risk.

## 3. Sex-Specific and Life Course Vulnerabilities

### 3.1. Adolescence: Critical Window of HPG Maturation

Adolescence represents a critical developmental window characterized by progressive activation and maturation of the HPG axis [4]. The pubertal transition involves gradual increases in GnRH pulsatility, resulting in incremental increases in LH and FSH secretion and the initiation of gonadal steroid production. This developmental process is exquisitely sensitive to perturbations, and disruptions during this window may result in persistent consequences for HPG axis function and reproductive health [21]. Adolescents exposed to chronic opioids face particular vulnerability during this developmental stage. Opioid-induced suppression of GnRH during critical pubertal windows may result in delayed pubertal progression [3]. Additionally, emerging evidence suggests that developmental opioid exposure may reprogram HPG axis function, resulting in persistent endocrine abnormalities even after opioid discontinuation [22]. Young women with adolescent-onset chronic opioid exposure may therefore face lifelong reproductive health consequences, including reduced fertility, altered menstrual function, and accelerated reproductive aging [21]. Quantitative human data in this age group remain extremely limited. Current concerns are based largely on extrapolation from preclinical models and from adult prevalence estimates of opioid-induced hypogonadism, which range from 15% to 63% across studies [10,11].

### 3.2. Reproductive Years: Menstrual Dysfunction and Fertility Implications

For women of reproductive age, opioid-induced HPG suppression can manifest as menstrual irregularity, anovulation, and impaired fertility. The systematic review by Wersocki et al. [16], the most comprehensive review of reproductive effects in women on long-term prescribed opioids, reported amenorrhea in 23 to 71% of women receiving oral or intrathecal opioids and decreased libido in 61 to 100%. The clinical study by Daniell [15] documented significantly lower levels of total testosterone, free testosterone, estradiol, LH, FSH, and DHEAS in women consuming oral or transdermal opioids compared to controls. LH and FSH values averaged 30% lower in premenopausal and 70% lower in postmenopausal opioid consumers. The dose-response relationship is also clinically meaningful: hypogonadism prevalence rises substantially at daily doses exceeding 100 morphine milligram equivalents (MME), and effects on the HPG axis can appear within weeks of initiating sustained-release opioid formulations [10,15]. Suppressed LH and FSH prevent ovulation, causing amenorrhea or oligomenorrhea [8].

However, opioid-induced reproductive dysfunction extends beyond absent cycles. Women can maintain regular menstruation while experiencing luteal phase insufficiency from a blunted LH surge and inadequate progesterone production [4]. This reproductive dysfunction carries profound clinical consequences. Women on chronic opioid therapy seeking conception face markedly reduced pregnancy rates, yet clinicians lack clear guidance on managing the intersection of opioid use disorder treatment and fertility goals [2,7].

### 3.3. Pregnancy: Opioid Exposure and Fetal/Neonatal Endocrine Disruption

Pregnancy presents a distinctive period of vulnerability for both the pregnant female and the developing fetus, and the endocrine consequences of chronic opioid exposure during this stage span both maternal and offspring. At the maternal level, opioid-induced endocrine dysfunction may worsen during pregnancy, with potential contributions to pregnancy complications including altered placental function, though the maternal endocrine trajectory during opioid-maintained pregnancies remains incompletely characterized [7].

At the fetal level, animal models have demonstrated that prenatal opioid exposure produces lasting alterations in HPG and HPA axis development. These include altered gonadal development in both sexes, with consequences that persist into postnatal life and adulthood [22]. However, human longitudinal data are scarce, and findings from rodent models cannot yet be extrapolated directly to humans. Prospective human studies examining long-term endocrine and reproductive outcomes in children prenatally exposed to opioids remain limited. While longitudinal studies have tracked neurodevelopmental and behavioral consequences of prenatal opioid exposure, endocrine outcomes have been largely unstudied [23]. Prenatal opioid exposure is also associated with neonatal opioid withdrawal syndrome (NOWS). NOWS involves not only acute withdrawal manifestations but dysregulation of stress-responsive neuroendocrine systems in the neonate, with long-term implications for HPA axis programming that are not yet established [7]. This constitutes yet another one of the most critically underinvestigated areas in the field.

### 3.4. Perimenopause and Menopause 

The menopausal transition represents another life stage with particular relevance to opioid-induced endocrine dysfunction. As women enter perimenopause and approach menopause, natural age-related declines in ovarian hormone production occur. For women with prior opioid-induced ovarian suppression or altered HPG axis function, the distinction between opioid-induced and age-related endocrine changes becomes difficult to discern [24]. Furthermore, perimenopause is characterized by heightened HPA axis reactivity and increased susceptibility to mood and anxiety disorders [25]. The addition of opioid-induced HPA axis dysregulation during this vulnerable window may significantly exacerbate psychiatric morbidity. Additionally, women entering menopause with prior opioid-induced hypogonadism may experience more severe vasomotor and neuropsychiatric menopausal symptoms compared to naturally postmenopausal women without prior endocrine disruption [26]. In a matched cohort study using the U.K. Clinical Practice Research Datalink, Richardson et al. [24] reported that women on long-term opioids had a significantly elevated risk of menopause before age 45 compared to matched non-opioid controls. The magnitude of this effect underscores that opioid-related reproductive aging is not subtle and warrants explicit counseling at the time of opioid initiation. Table 1 summarizes opioid-associated hormonal changes across all life-course stages discussed in this section.

## 4. Epidemiology and Prevalence

### 4.1. Sex Differences in Opioid-Induced Endocrine Dysfunction

Epidemiological studies comparing opioid-induced endocrine dysfunction across sexes are severely limited by the underrepresentation of women in existing research. The de Vries et al. [10] systematic review and meta-analysis, the most methodologically comprehensive to date, reported a 63% prevalence of hypogonadism in opioid-using men drawn from studies in which women comprised less than 0.5% of the total pooled participant sample. This sex imbalance means that a clinically reliable female-specific prevalence estimate for opioid-induced hypogonadism does not yet exist from meta-analytic evidence. Table 2 summarizes female representation across the major studies and systematic reviews of opioid-induced endocrine dysfunction. The female-specific evidence base is sparse, derived primarily from three key studies. Daniell [15] documented hypogonadotropic hypogonadism and suppressed gonadotropins in women on sustained-action opioids. Bawor et al. [27] examined 231 patients on methadone maintenance therapy (100 women). In striking contrast to the robust dose-dependent testosterone suppression observed in men, they found no significant effect of methadone on serum testosterone in women relative to non-opioid controls (*p* = 0.441). A subsequent systematic review by Bawor et al. [28] confirmed that opioid effects on testosterone differ between sexes, with effects in women showing greater variability and remaining less thoroughly characterized than those in men.

### 4.2. Prevalence in Chronic Pain Populations

Among individuals prescribed chronic opioids for pain management, the prevalence of menstrual and endocrine dysfunction varies widely depending on factors such as opioid dose, formulation, duration of exposure, and measurement methodology. Studies report hypogonadism prevalence ranging from 15 to 63% in chronic pain populations, with higher prevalence consistently associated with higher morphine milligram equivalent daily doses [10,11]. Specifically, in the de Vries et al. meta-analysis, the pooled odds of hypogonadism rose with increasing daily MME, with the highest prevalence (above 60%) reported in cohorts on daily doses exceeding 100 MME or on long-acting formulations [10,11]. Among women specifically, Wersocki et al. [16] found that long-term opioid use was associated with a statistically significant increased risk of altered menstruation and of earlier menopause, providing large-scale epidemiological support for the clinical observations from smaller hormonal studies [16,24].

### 4.3. Prevalence in Opioid Use Disorder

Individuals with opioid use disorder may experience particularly severe endocrine dysfunction, reflecting higher cumulative opioid exposure and often more variable dosing patterns compared to individuals prescribed opioids for medical reasons [28]. Studies of individuals with opioid use disorder reveal high prevalence of hypogonadism, amenorrhea, and other endocrine abnormalities [7]. Additionally, the heterogeneity of drugs used in opioid use disorder (varying potency, pharmacokinetics, and impurity profiles) can result in variable endocrine effects compared to prescribed opioids [2].

## 5. Clinical Manifestations and Health Outcomes

### 5.1. Hypogonadism and Sexual Health 

Opioid-induced hypogonadism in women produces clinical manifestations across multiple physiological systems. Low circulating estradiol results in diminished vaginal lubrication, decreased sexual desire and arousal, and anorgasmia [15,16]. These changes affect quality of life and intimate relationships. They can also contribute to treatment non-adherence when individuals discontinue opioids due to sexual dysfunction [11]. Additionally, opioid-induced hypogonadism contributes to reduced bone density, increased fracture risk, and impaired wound healing. These consequences disproportionately affect women given the already elevated fracture risk in postmenopausal populations [2,29]. Coluzzi et al. [29] identified three mechanistic pathways by which opioids impair bone metabolism: direct effects on osteoblasts, indirect effects via hypogonadism and reduced gonadal steroid output, and effects on central neuroendocrine regulators of bone turnover. These overlapping pathways help explain why bone loss in chronic opioid users can occur even when sex steroid replacement is initiated late in the course of exposure.

### 5.2. Mood and Cognitive Effects

Beyond direct neuroendocrine effects, opioid-induced hypogonadism and HPA axis dysregulation contribute to mood and cognitive dysfunction [10]. Low estrogen levels are associated with increased depression and anxiety risk, particularly in vulnerable populations [25]. The mechanisms are multifactorial, involving estrogen’s effects on serotonergic and GABAergic neurotransmitter systems, as well as its role in hippocampal synaptic plasticity and neurogenesis. Women with opioid-induced hypogonadism frequently present with depression, anxiety, and cognitive fog. These symptoms may be partially attributable to estrogen deficiency and may not fully resolve with standard antidepressant or anxiolytic therapy [11].

### 5.3. Metabolic Health

As discussed earlier, opioid-induced endocrine disruption produces metabolic consequences such as insulin resistance, visceral adiposity, and dyslipidemia [2]. Some women report weight gain during periods of chronic opioid use. The extent to which this is attributable to opioid-induced hypogonadism, as opposed to other factors such as sedentary lifestyle, dietary changes, and direct metabolic effects of opioids, has not been quantitatively established [11]. The development of metabolic dysfunction is hypothesized to create a bidirectional feedback loop. Obesity itself dysregulates the HPG axis, and metabolic syndrome is associated with further alterations in adrenal and thyroid function, which may compound opioid-induced endocrine disruption [20].

### 5.4. Quality of Life and Functional Outcomes

Collectively, the endocrine, metabolic, and psychiatric consequences of chronic opioid use produce profound impairment in quality of life and functional capacity. Women report diminished energy, reduced exercise capacity, impaired occupational and social functioning, and decreased life satisfaction [2,11]. These outcomes may not be adequately captured by standard pain intensity scales or addiction severity measures, underscoring the need for more comprehensive assessments in individuals with chronic opioid exposure.

## 6. Diagnostic and Clinical Assessment Challenges

### 6.1. Symptom Overlap and Attribution

A fundamental challenge in recognizing opioid-induced endocrine dysfunction in women is the substantial overlap between endocrine symptoms and opioid use disorder comorbidities. Fatigue, mood disturbance, sexual dysfunction, and weight change are common presentations of depression, anxiety, and chronic pain, conditions that co-occur at high rates with chronic opioid exposure [11]. This overlap creates systematic opportunities for misattribution, with physicians attributing endocrine related manifestations to primary psychiatric or pain conditions rather than investigating underlying hormonal abnormalities. Estimates suggest that only approximately 10% of patients with OIAI will ever receive a correct diagnosis. Two-thirds are misdiagnosed prior to accurate identification, and the median diagnostic delay exceeds six months [19]. These figures speak to a systemic failure of clinical recognition that begins with inadequate awareness of opioid-induced endocrine dysfunction as a distinct clinical entity [2].

### 6.2. Menstrual Cycle Confounding

For women, assessment of reproductive and endocrine function is complicated by menstrual cycle dependent variation in estradiol, progesterone, LH, and FSH. Standard laboratory reference ranges are typically derived from specific cycle-phase sampling windows, but opioid-induced suppression may manifest as disrupted cycle dynamics rather than low hormone levels, and opioid-induced menstrual irregularity may itself prevent standardized phase specific sampling [15,16]. This creates a diagnostic challenge. Physicians must interpret hormone levels in the context of menstrual history and recognize that abnormal cycle dynamics, not just abnormal hormone levels, indicate endocrine disruption.

### 6.3. Lack of Clinical Guidelines

Despite the clinical burden of opioid-induced endocrine dysfunction, formalized screening and management guidelines have been quite slow to develop. The Endocrine Society’s 2024 Scientific Statement, “Exogenous Opioids and the Human Endocrine System” [2], represents the most recent effort to systematize knowledge in this area and identifies the absence of sex-stratified data and clinical guidelines as priority gaps. The Society of Obstetricians and Gynaecologists of Canada (SOGC) has also published Guideline No. 443a, “Opioid Use Throughout Women’s Lifespan,” which specifically addresses endocrine and reproductive consequences of opioid exposure across the female lifespan and provides preliminary clinical guidance, though the evidence base supporting its recommendations is largely expert consensus rather than trial derived [30]. Despite these advances, most physicians lack both awareness of opioid-induced endocrine dysfunction as a discrete clinical entity and practical frameworks for its assessment and management [2,19].

## 7. Management and Treatment Considerations

### 7.1. Opioid Reduction and Tapering

The most effective strategy for mitigating opioid-induced endocrine dysfunction is reduction or discontinuation of opioid exposure [2,11]. Animal studies demonstrate that cessation of opioid exposure results in rapid restoration of GnRH secretion and gonadotropin levels [3]. Limited human data support partial reversibility of opioid-induced adrenal insufficiency. Li et al. [31] followed patients with OIAI who tapered or discontinued opioids: 70% recovered adrenal function, while the remaining 30% did not. Clinical experience and limited human data therefore suggest that endocrine recovery following prolonged opioid exposure may be incomplete or protracted, particularly in individuals with adolescent onset exposure or very high cumulative lifetime doses [22]. For individuals with chronic pain on opioid therapy, opioid tapering must be undertaken with appropriate clinical supervision and consideration of pain management alternatives. Abrupt opioid discontinuation may precipitate withdrawal symptoms and pain exacerbation, necessitating gradual dose reduction. During the tapering process, individuals should be monitored for resolution of endocrine-related symptoms and, if indicated, reassessment of hormone levels [2].

### 7.2. Medication for Opioid Use Disorder (OUD)

For individuals with OUD, the choice of medication for opioid use disorder carries endocrine implications that are not yet fully established but deserve clinical consideration. Methadone, a full mu opioid agonist with high receptor occupancy and a long half-life, has been associated with greater suppression of gonadotropins and sex hormones than buprenorphine in comparative studies [28]. Buprenorphine’s partial agonist profile and ceiling effect at mu receptors may translate into less severe HPG suppression, which has particular relevance for women with fertility goals or significant hypogonadism-related symptoms [28,32]. The available comparative evidence is not yet sufficient to support firm clinical recommendations. Medication selection should continue to be guided primarily by addiction treatment efficacy and patient-specific factors. Endocrine considerations should be incorporated into shared decision-making for women with fertility or reproductive health concerns.

### 7.3. Hormone Replacement Therapy (HRT)

For females unable to discontinue opioids or with incomplete endocrine recovery following opioid cessation, hormone replacement therapy may offer symptomatic benefit [11]. Estrogen replacement can address hypogonadism-related symptoms such as sexual dysfunction, mood disturbance, and bone loss [25]. However, evidence for HRT efficacy in opioid-induced hypogonadism is limited, and long-term safety remains inadequately characterized [2]. Importantly, HRT addresses symptomatic manifestations of hypogonadism but does not reverse the underlying endocrine dysfunction or necessarily improve fertility in women seeking pregnancy. Additionally, estrogen replacement may carry risks like increased thromboembolism in individuals with immobility or other thrombotic risk factors, particularly in those with concurrent opioid-related complications [11].

The risk profile of HRT in opioid-exposed women warrants particular attention. Chronic opioid use is associated with reduced physical activity, higher rates of smoking, and elevated body mass index in many cohorts. Each of these factors independently raises venous thromboembolism (VTE) risk, and adding systemic estrogen may compound that risk [2,11]. The 2022 North American Menopause Society position statement advises that transdermal rather than oral estradiol is generally preferred in women with elevated thromboembolic risk. Transdermal preparations bypass first-pass hepatic metabolism and do not significantly elevate clotting factor synthesis [33]. Progesterone or a progestin should be added in women with an intact uterus to protect against endometrial hyperplasia. The decision to initiate HRT should be individualized, made in consultation with endocrinology or reproductive medicine, and revisited annually given evolving risk profiles.

### 7.4. Fertility and Reproductive Planning

Fertility management in women with opioid-induced endocrine dysfunction is one of the most clinically complex and under-evidenced areas in this field. The available data support several practical principles.

First, opioid-induced anovulation is generally reversible with opioid reduction or discontinuation. Restoration of ovulatory cycles typically occurs within weeks to a few months in preclinical models and in limited human case series [3,15]. However, women with adolescent-onset exposure, very high cumulative lifetime doses, or more than five years of continuous high-dose therapy may experience incomplete recovery. No reliable biomarker predicts which patients will recover normal HPG function [2,22].

Second, for women on medications for opioid use disorder (MOUD) who wish to conceive, transition from methadone to buprenorphine may be considered on the basis of the suggestive evidence that buprenorphine produces less HPG suppression [28,34]. This recommendation remains tentative rather than established. Medication transitions during preconception planning should be undertaken only in coordination with addiction medicine specialists, given the substantial risks of relapse and overdose associated with treatment instability [32,35].

Third, women of reproductive age on chronic opioids should receive explicit fertility counseling at the time of opioid initiation and at least annually thereafter. Counseling should address the expected effects of opioids on menstrual function and fertility, the reversibility of these effects, the importance of contraception for women who do not wish to conceive (since anovulation is incomplete and unintended pregnancy remains possible), and the options available for women who wish to conceive while on opioid therapy [7,30].

Fourth, assisted reproductive technologies (ART) may be considered for women with persistent infertility despite opioid optimization, but should not be pursued as a substitute for addressing the underlying endocrine disruption. There are no published prospective trials of ART outcomes specifically in opioid-exposed women, and clinical decisions in this area rest on expert consensus rather than trial evidence [2].

Finally, contraceptive counseling deserves equal emphasis. Women on chronic opioids who do not wish to conceive should be offered the full range of contraceptive options. Long-acting reversible contraceptives (LARCs) are generally preferred because their efficacy does not depend on adherence, which can be irregular during periods of active opioid use or treatment transitions [30,35].

### 7.5. Lifestyle and Supportive Interventions

Interventions including regular physical activity, adequate nutrition, stress reduction, and sleep hygiene may help mitigate endocrine and metabolic consequences of chronic opioid use [2]. Physical activity in particular has demonstrated benefits for insulin sensitivity, visceral adiposity, and mood in individuals with opioid use disorder. However, the specific effects of these interventions on opioid-induced endocrine dysfunction have not been systematically evaluated.

## 8. Research Gaps and Future Directions

Despite growing recognition of opioid-induced endocrine dysfunction, fundamental mechanistic and epidemiological questions remain inadequately addressed in women. Several research priorities are particularly urgent.

Opioid-induced hypogonadism in women involves multiple potential mechanisms whose relative importance remains undefined. Central opioid receptor signaling and direct ovarian effects likely both contribute, yet their relative roles are unclear [4,10]. Additionally, the sources of variable individual susceptibility and potential protective mechanisms have not been characterized. Large prospective cohort studies characterizing the natural history of opioid-induced endocrine dysfunction in women are urgently needed. These studies should prospectively evaluate endocrine function across the lifespan, assessing recovery trajectories following opioid cessation, identifying modifiable and non-modifiable risk factors, and incorporating sex-stratified analyses in existing opioid research cohorts [2,16].

Particular attention should be directed toward the consequences of opioid exposure during critical developmental windows, including adolescence, pregnancy, and early postnatal life, where effects may be most durable and most likely to produce intergenerational consequences. The possibility of epigenetic programming of HPG and HPA axis function by developmental opioid exposure warrants investigation within the xenobiotic endocrine disruption framework [22,23].

Randomized controlled trials comparing management strategies, including opioid tapering approaches, differential medication effects on endocrine outcomes, HRT protocols, fertility interventions, and lifestyle-based strategies, should evaluate endocrine outcomes alongside traditional pain and addiction endpoints. The current complete absence of this evidence for any endocrine management approach in opioid exposed women is untenable given the scope of our problem [2].

Lastly, the development and validation of sex-specific screening tools and clinical diagnostic criteria for opioid-induced endocrine dysfunction in women is needed. Such tools must account for menstrual cycle-dependent hormone variation, be feasible in primary care and addiction medicine settings, and incorporate validated outcome measures for sexual function, mood, and reproductive symptoms alongside biochemical markers.

## 9. Clinical Implications and Recommendations

Physicians caring for women on chronic opioid therapy or in treatment for opioid use disorder should incorporate routine endocrine screening into clinical practice. Based on existing expert consensus [2,11,30] and adapted to address sex-specific considerations, we propose the screening framework outlined in Table 3.

Baseline assessment should be performed within three months of opioid initiation, or at the next clinical encounter for women already on long-term therapy [2,30]. Initial evaluation should include a structured symptom inventory (menstrual regularity, libido, sexual function, mood, fatigue, weight trajectory, and vasomotor symptoms) together with a biochemical panel: morning (08:00–09:00) serum cortisol, estradiol, LH, FSH, prolactin, total testosterone, DHEAS, and TSH with free T4 [36,37]. The preference for early-morning cortisol sampling reflects the diurnal peak of HPA axis activity and is the timing endorsed by the Endocrine Society for screening adrenal insufficiency [36]. In cycling women, sex-steroid sampling should ideally occur in the early follicular phase (cycle days 2–5), when baseline FSH and estradiol levels are most interpretable for ovarian function assessment [37]. When cycles are irregular, random sampling should be interpreted alongside menstrual history rather than against standardized phase-specific reference ranges. Bone mineral density (DXA) should be considered at baseline in women with ≥12 months of opioid exposure, particularly at doses ≥50 morphine milligram equivalents (MME) per day, consistent with International Society for Clinical Densitometry indications for secondary osteoporosis screening in adults at elevated fracture risk, and repeated every two years if abnormal [29,38].

Follow-up frequency should be calibrated to clinical risk rather than prescribed at fixed monthly intervals. We suggest annual reassessment of the symptom inventory and biochemical panel in stable patients, with more frequent (every three to six months) evaluation following opioid dose escalation, new symptom onset, or initiation of hormone replacement therapy. Morning cortisol below 5 μg/dL, or a clinical picture suggestive of adrenal insufficiency, should prompt confirmatory dynamic testing (ACTH stimulation) and endocrinology referral [17,19,31].

The clinical rationale for this approach rests on three observations. First, opioid-induced endocrine dysfunction is highly prevalent, with hypogonadism reported in 15–63% of chronic opioid users and adrenal insufficiency in 15–24% [10,11]. Second, the symptoms are non-specific and routinely misattributed: fewer than 10% of patients with opioid-induced adrenal insufficiency receive a correct diagnosis, and the median diagnostic delay exceeds six months [19,31]. Third, the downstream consequences such as infertility, osteoporotic fracture, adrenal crisis, and untreated depression carry substantial morbidity and cost, much of which is preventable with early identification. The biochemical panel described above is inexpensive, widely available, and far less costly than the downstream care of unrecognized fracture, infertility evaluation, or emergency adrenal crisis management. Routine screening therefore represents a high-value clinical intervention rather than an additional diagnostic burden.

Such assessment is best delivered within multidisciplinary care frameworks that integrate pain management, addiction treatment, reproductive health, and endocrinology, given the breadth of systems affected and the frequency with which symptoms are misattributed to psychiatric or pain conditions rather than recognized as endocrine in origin. Table 4 summarizes the recommended roles of each clinical specialty in the identification, monitoring, and management of opioid-induced endocrine dysfunction in women.

Equally important is the incorporation of informed consent and counseling into routine care. Women initiating chronic opioid therapy, particularly adolescents and those of reproductive age, should be counseled about potential endocrine and reproductive consequences before treatment begins, and those already on opioid therapy should be made aware that hormonal dysfunction may be contributing to symptoms they might otherwise not associate with their medication [7,11]. Also, prescribing practices that minimize cumulative opioid exposure through regular reassessment of necessity and use of non-opioid alternatives represent an important strategy for reducing endocrine harm in this population [2].

Based on the evidence reviewed above, we propose the following specific clinical recommendations for the care of women on chronic opioid therapy:

1. Screen all women initiating chronic opioid therapy at baseline (within 3 months of initiation) with a structured symptom inventory and the biochemical panel detailed in Table 3.
2. Reassess annually in stable patients, and every 3–6 months following dose escalation, new symptoms, or initiation of hormone replacement.
3. Provide explicit fertility and contraceptive counseling at opioid initiation for all women of reproductive age, and reassess reproductive goals annually.
4. Obtain a baseline DXA scan in women with ≥12 months of opioid exposure or daily doses ≥50 MME, and repeat every two years if abnormal.
5. Consider transition from methadone to buprenorphine in women with significant hypogonadism-related symptoms or active fertility goals, in coordination with addiction medicine.
6. Investigate adrenal insufficiency with morning cortisol and, where indicated, ACTH stimulation testing in any opioid-exposed woman presenting with fatigue, hypotension, hyponatremia, or unexplained weight loss.
7. Counsel women initiating chronic opioids about the expected endocrine and reproductive consequences of treatment as part of informed consent.

## 10. Conclusions

Chronic opioid exposure produces profound disruption of women’s endocrine function, with consequences spanning reproductive health, metabolic function, mood, bone health, and quality of life. The mechanisms underlying this disruption involve opioid-mediated suppression across the HPG, HPA, and HPT axes through converging and interrelated pathways. Sex-specific vulnerabilities across the lifespan, from adolescent HPG maturation through the menopausal transition, create distinct windows of heightened risk that may produce lasting and potentially intergenerational consequences.

Despite this burden, opioid-induced endocrine dysfunction remains strikingly underrecognized in clinical practice, underrepresented in research cohorts, and starkly absent from most clinical guidelines. The most comprehensive meta-analytic evidence base in this field was derived from a sample that was 99.5% male, and female-specific guidance is only now beginning to emerge in the clinical space. Addressing this gap requires coordinated efforts across addiction medicine, endocrinology, reproductive health, and epidemiology, with a commitment to centering women’s endocrine needs in research design from the outset. As the opioid crisis continues to affect millions globally, attention to the full spectrum of opioid-induced toxicity is essential to true patient-centered care. Women deserve clinical attention and research investment that explicitly acknowledges their unique endocrine vulnerabilities, rather than continuing to extrapolate guidance from an evidence base built predominantly on male experience.

## Figures and Tables

**Table 1:** Summary of opioid-associated hormonal changes across life-course stages in women. ↓, opioid-driven decrease relative to age-matched controls; ↓↓, compounded decrease reflecting opioid suppression layered onto declining estrogen due to aging; ↑, paradoxical increase due to opioid inhibition of hypothalamic dopaminergic tone; –, insufficient data.

Life Stage	Estradiol	LH/FSH	Cortisol	Prolactin	Clinical Risks
Adolescence	↓	↓	↓	↑	Pubertal delay, impaired bone accrual
Reproductive Years	↓	↓	↓	↑	Amenorrhea, infertility, bone loss
Pregnancy	↓	–	↓	–	Fetal HPG programming, NOWS
Perimenopause	↓↓	↓	↓	↑	Accelerated menopause onset, vasomotor overlap
Post-menopause	↓↓	↓	↓	–	Persistent bone loss, metabolic syndrome

**Table 2:** Female representation in major studies and systematic reviews examining opioid-induced endocrine dysfunction. The near-total absence of female participants in pooled analyses limits the generalizability of prevalence estimates to women.

Study	Total Sample	Female Participants	Female-Specific Results Reported?
de Vries et al., 2020 [10] (systematic review and meta-analysis)	2645	Less than 0.5%	No
Bawor et al., 2015 [28] (systematic review, testosterone suppression)	1188	0%	No
Daniell, 2008 [15] (observational cohort, women only)	57	100%	Yes
Wersocki et al., 2017 [16] (systematic review, women only)	Mixed; small cohorts	100%	Yes
Fountas et al., 2020 [11] (narrative review)	Review only	Not reported	No
Karavitaki et al., 2024 [2] (Endocrine Society scientific statement)	Review only	Partially addressed	Identifies female data gap explicitly

**Table 3:** Proposed screening framework for opioid-induced endocrine dysfunction in women. Frequencies are suggested defaults and should be adjusted based on opioid dose, duration, symptoms, and individual risk factors. MME, morphine milligram equivalents; DXA, dual-energy X-ray absorptiometry; DHEAS, dehydroepiandrosterone sulfate; ACTH, adrenocorticotropic hormone.

Domain	Recommended Assessment	Suggested Frequency
Symptom inventory	Menstrual regularity, libido, sexual function, mood, fatigue, weight trajectory, vasomotor symptoms	Baseline; then annually, or every 3–6 months after dose change
HPG axis	Estradiol, LH, FSH, total testosterone, prolactin (cycle days 2–5 if cycling)	Baseline; annually; with new symptoms
HPA axis	Morning (08:00–09:00) serum cortisol; ACTH stimulation if cortisol < 5 μg/dL or symptoms suggest insufficiency	Baseline; annually; before surgery or major stressor
HPT axis	TSH and free T4	Baseline; annually
Adrenal androgens	DHEAS	Baseline; repeat if symptoms of androgen deficiency
Bone health	DXA scan	Baseline if ≥12 months exposure or ≥50 MME/day; every 2 years if abnormal
Fertility counseling	Discussion of reproductive goals, contraception, and impact of opioid exposure on fertility	At initiation; annually for reproductive-age women

**Table 4:** Recommended roles of clinical specialties in the identification, monitoring, and management of opioid-induced endocrine dysfunction in women [2,11,30]. MOUD, medications for opioid use disorder; OIAI, opioid-induced adrenal insufficiency; HPT, hypothalamic–pituitary–thyroid; HPA, hypothalamic–pituitary–adrenal.

Clinical Specialty	Role in Opioid-Induced Endocrine Dysfunction
Primary Care	First point of contact; screen for menstrual, sexual, mood, and weight changes; initiate hormone panel and coordinate referrals
Pain and Addiction Medicine	Assess opioid necessity; optimize MOUD selection; consider dose reduction or non-opioid alternatives
Endocrinology	Confirm diagnosis of OIAI, hypogonadism, or HPT disruption; oversee hormone replacement and dynamic testing
Reproductive Medicine and Gynecology	Manage fertility planning, ovarian reserve assessment, and perimenopause or menopause care
Psychiatry or Psychology	Distinguish HPA-driven mood and cognitive symptoms from primary psychiatric disorder; support opioid tapering
Pharmacy	Review CYP450-mediated interactions between opioids and hormone therapies; support medication reconciliation

## Data Availability

No new data were created or analyzed in this study. Data sharing is not applicable to this article.

## Abbreviations

The following abbreviations are used in this manuscript:

ACTH	Adrenocorticotropic hormone
ART	Assisted reproductive technologies
CRH	Corticotropin-releasing hormone
DHEAS	Dehydroepiandrosterone sulfate
DXA	Dual-energy X-ray absorptiometry
FSH	Follicle-stimulating hormone
GABA	Gamma-aminobutyric acid
GnRH	Gonadotropin-releasing hormone
HPA	Hypothalamic–pituitary–adrenal
HPG	Hypothalamic–pituitary–gonadal
HPT	Hypothalamic–pituitary–thyroid
HRT	Hormone replacement therapy
KNDy	Kisspeptin/neurokinin B/dynorphin
LARC	Long-acting reversible contraceptive
LH	Luteinizing hormone
MME	Morphine milligram equivalents
MOUD	Medications for opioid use disorder
NOWS	Neonatal opioid withdrawal syndrome
OIAI	Opioid-induced adrenal insufficiency
OUD	Opioid use disorder
PET	Positron emission tomography
PVN	Paraventricular nucleus
SOGC	Society of Obstetricians and Gynaecologists of Canada
T4	Thyroxine
TRH	Thyrotropin-releasing hormone
TSH	Thyroid-stimulating hormone
VTE	Venous thromboembolism
